# Retraction: MiR-375 Is Epigenetically Downregulated by HPV-16 E6 Mediated DNMT1 Upregulation and Modulates EMT of Cervical Cancer Cells by Suppressing lncRNA MALAT1

**DOI:** 10.1371/journal.pone.0224167

**Published:** 2019-10-15

**Authors:** The PLOS ONE Editors

Concerns have been raised that in Fig 5D of this article [1] several clusters of cells appear to be duplicated within and across panels. In addition, the Data Availability statement for this article reads, “All relevant data are within the paper and its Supporting Information file,” but primary data were not included within the article.

The corresponding author noted that the original data supporting the results in this article are no longer available, and that the authors have been unable to replicate some of the reported results.

In light of these issues, the *PLOS ONE* Editors retract this article.

The authors did not reply to the retraction notification.

## References

[1] Liu S, Song L, Yao H, Zhang L, Xu D, Gao F, et al (2016) MiR-375 Is Epigenetically Downregulated by HPV-16 E6 Mediated DNMT1 Upregulation and Modulates EMT of Cervical Cancer Cells by Suppressing lncRNA MALAT1. PLoS ONE 11(9): e0163460 10.1371/journal.pone.0163460 27658300 PMC5033370

